# Systematic Review of the Association of the Hospital Frailty Risk Score with Mortality in Patients with Cerebrovascular and Cardiovascular Disease

**DOI:** 10.2174/011573403X276647240217112151

**Published:** 2024-02-29

**Authors:** Balamrit Singh Sokhal, Sowmya Prasanna Kumar Menon, Charles Willes, Nadia Corp, Andrija Matetić, Christian Mallen, Mamas Mamas

**Affiliations:** 1 School of Medicine, Keele University, Keele, Staffordshire, ST5 5BG, United Kingdom;; 2 Keele Cardiovascular Research Group, Centre for Prognosis Research, Keele University, Keele, Staffordshire, ST5 5BG, United Kingdom;; 3 Department of Cardiology, University Hospital of Split, Split 21000, Croatia.

**Keywords:** Frailty, cardiovascular disease, systematic review, hospital frailty risk score, mortality, cerebrovascular disease, atrial fibrillation

## Abstract

**Background:**

There is limited systematic data on the association between the Hospital Frailty Risk Score (HFRS) and characteristics and mortality in patients with cerebrovascular and cardiovascular disease (CVD). This systematic review aimed to summarise the use of the HFRS in describing the prevalence of frailty in patients with CVD, the clinical characteristics of patients with CVD, and the association between frailty on the likelihood of mortality in patients with CVD.

**Methods:**

A systematic literature search for observational studies using terms related to CVD, cerebrovascular disease, and the HFRS was conducted using 6 databases in accordance with the Preferred Reporting Items for Systematic Reviews and Meta-Analyses guidelines. Studies were appraised using the Newcastle-Ottawa Scale (NOS).

**Results:**

Seventeen observational studies were included, all rated ‘good’ quality according to the NOS. One study investigated 5 different CVD cohorts (atrial fibrillation (AF), heart failure (HF), hypotension, hypertension, and chronic ischemic heart disease), 1 study investigated 2 different CVD cohorts (AF and acute myocardial infarction (AMI)), 6 studies investigated HF, 3 studies investigated AMI, 4 studies investigated stroke, 1 study investigated AF, and 1 study investigated cardiac arrest. Increasing frailty risk category was associated with increased age, female sex, and non-white racial group across all CVD. Increasing frailty risk category is also associated with increased length of hospital stay, total costs, and increased odds of 30-day all-cause mortality across all CVD.

**Conclusions:**

The HFRS is an efficient and effective tool for stratifying frailty in patients with CVD and predicting adverse health outcomes.

## 1 INTRODUCTION

The average age of the population is increasing, so the number of patients at risk of frailty is rising [1-3]. Frailty is conceptually defined as a clinical syndrome characterized by loss of the body’s homeostatic mechanisms, resulting in susceptibility to stress and poor outcomes such as institutionalization and death [4].

Cardiovascular disease (CVD) is the largest cause of mortality and morbidity worldwide [5]. Frailty and CVD are related, with up to 60% of people with CVD associated with a three-fold increased risk of mortality from CVD [6, 7]. The underlying mechanisms are poorly understood. However, this could be due to a chronic inflammatory state, shared risk factors, and comorbidity [1, 8, 9]. Frail patients admitted to the hospital experience increased length of stay (LOS), total costs, and likelihood of all-cause mortality.

Various tools are available to stratify frailty in primary care and hospital settings [10-14]. One such tool is the Hospital Frailty Risk Score (HFRS), which quantifies frailty using 109 individually weighted comorbidities using the International Classification of Diseases (ICD) – 10^th^ edition codes. The score stratifies patients into 3 frailty risk categories based on a score of 0 to 99: Low (<5), intermediate (5-15), and high (>15). The score can be derived from electronic health record data, so the HFRS is less resource-intensive than other frailty assessment tools.

This systematic review and narrative synthesis aimed to summarise the currently published evidence about the use of the HFRS in describing the prevalence, clinical characteristics, and likelihood of adverse outcomes of patients with CVD, stratified by their frailty risk.

## 2 METHODS

### 2.1 Search Strategy and Study Selection

**Fig. (1):**
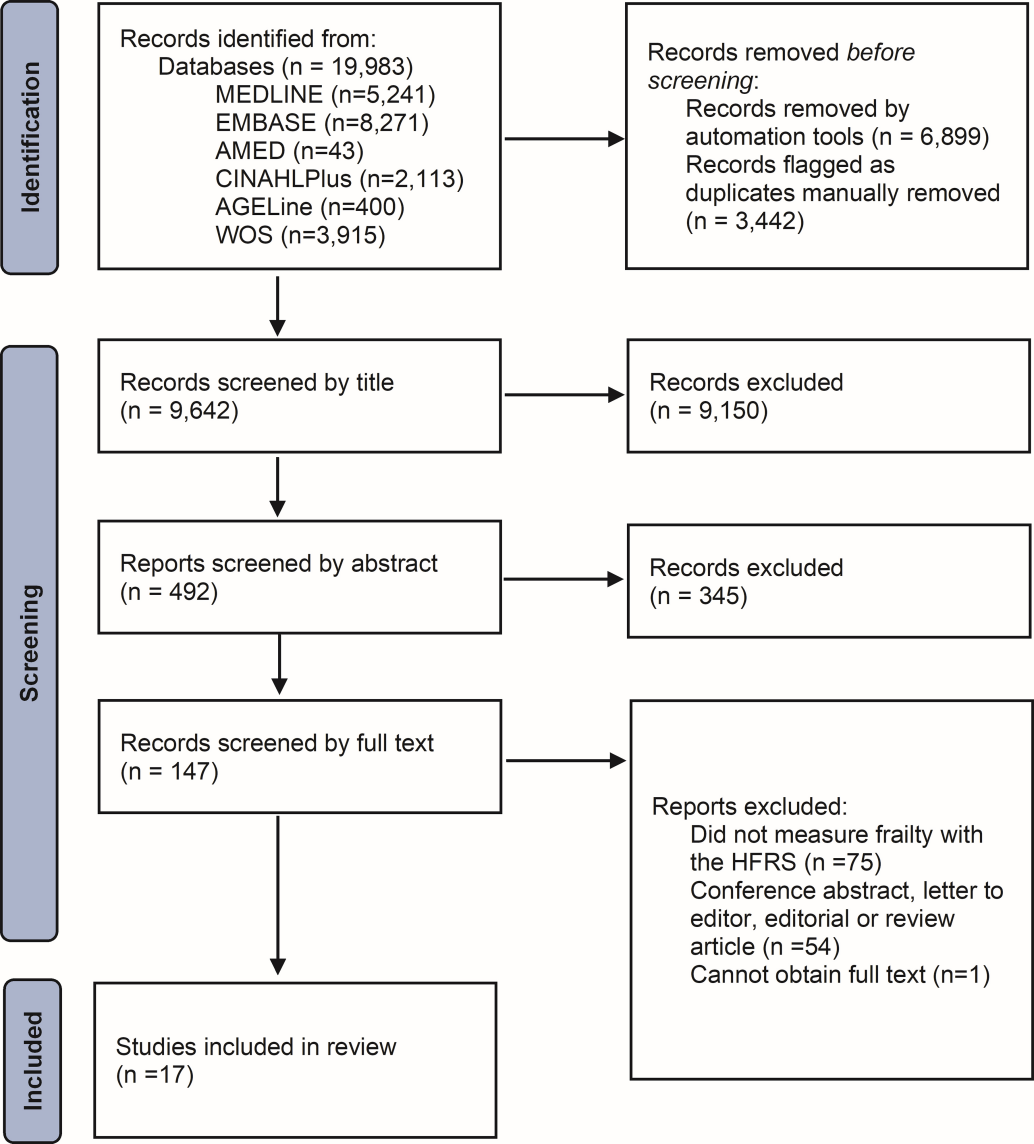
PRISMA flow diagram of the systematic review. **Abbreviations:** Cardiovascular Disease – CVD; HFRS – Hospital Frailty Risk Score; Web of Science – WOS.

The protocol for this review was pre-registered with the *International Prospective Register of Systematic Reviews* (PROSPERO, CRD42022371883). This review follows *Preferred Reporting Items for Systematic Reviews and Meta-Analyses* (PRISMA) guidance (Fig. **1**) [15]. MEDLINE (EBSCO), EMBASE (Ovid), AMED (EBSCO), CINAHLPlus (EBSCO), AGELine (EBSCO) and Web of Science from inception until November 2022 were comprehensively searched by one reviewer (BSS). The search strategy utilized both database subject headings and text word searching in the title and abstract using terms for “hospital frailty risk score” and “cardiovascular disease” (Appendix **1**).

### 2.2 Inclusion and Exclusion Criteria

Studies using the HFRS, investigation of prevalence, clinical characteristics, and/or outcomes of patients with CVD published in English were included. Study designs eligible for inclusion were randomized controlled trials and observational studies. There was no restriction on the time or the definition of CVD. Conference abstracts, research letters, editorials, case reports, case series, literature reviews, and other systematic review articles were excluded.

### 2.3 Screening and Data Extraction

All references were imported into a reference management software and de-duplicated by one reviewer (BSS). Following de-duplication, the same reviewer imported references into Rayyan and screened all titles against the inclusion criteria, excluding references that did not meet these criteria. Two reviewers (BSS and SPKM) independently screened the remaining abstracts and full texts against the inclusion criteria.

Two reviewers (BSS and SPKM) independently extracted data from full-text articles into pre-formed tables. The following data were extracted: Study characteristics (study design, study year, and country), patient characteristics (number and age of the participants stratified by frailty status, the prevalence and clinical characteristics of the participants stratified by frailty status), and study outcomes (length of stay, readmission rate, and all-cause mortality stratified by frailty status). Disagreements throughout the screening and data extraction process were resolved by discussion. A third independent reviewer provided a resolution (CW) where disagreements remained.

### 2.4 Quality Evaluation

The quality of each study was assessed independently by two reviewers (BSS and SPKM) using the *Newcastle-Ottawa Quality Assessment for Cohort Study* tool [16, 17], using 3 domains (selection, comparability, and outcome), each with multiple choice outcomes and a ‘star’ to represent quality. The selection domain assessed the representativeness and selection of the cohort and the ascertainment of whether the exposure was present at the start of the study. The comparability domain assessed the quality of adjustment for confounders. The outcome domain assessed how the outcome was ascertained, the length of follow-up, and the adequacy of follow-up. Finally, a grade of ‘good quality,’ ‘fair quality,’ or ‘poor quality’ was assigned.

### 2.5 Data Synthesis and Analysis

Given the variation of inclusion criteria, HFRS cut-off for analyses, and reporting of HFRS groups among studies, a meta-analysis was not conducted. The narrative synthesis was developed and reported in accordance with the *Synthesis Without Meta-analysis* (SWiM) guidance and the *Cochrane Handbook* [18, 19]. Results were grouped according to the CVD investigated in each study.

## 3 RESULTS

### 3.1 Study Characteristics

**Fig. (2):**
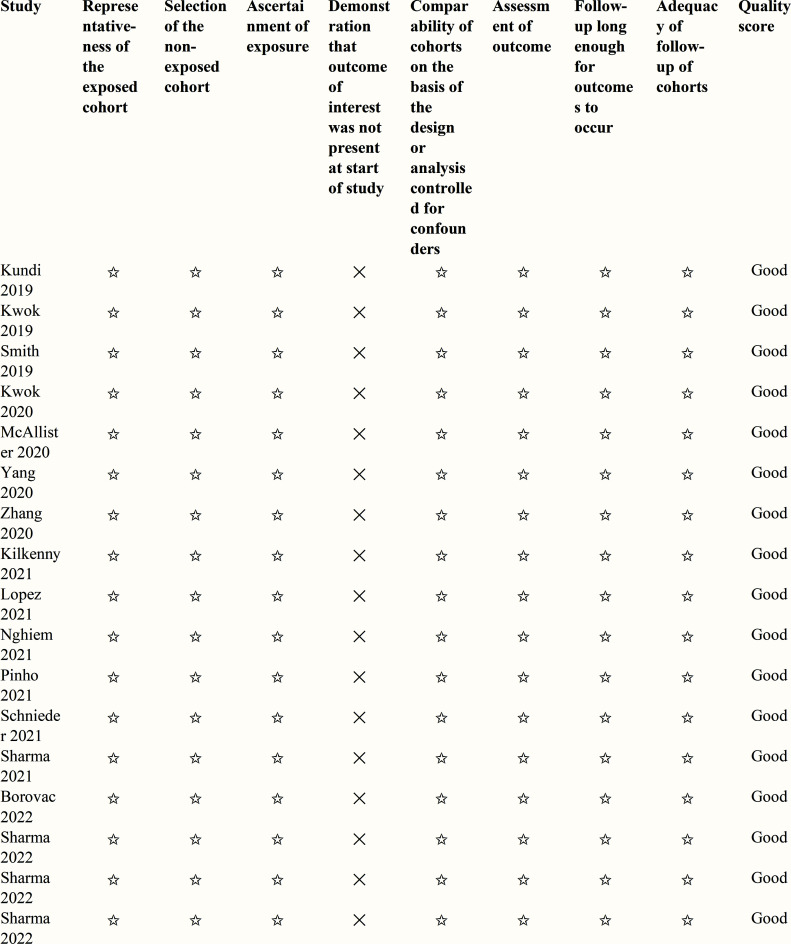
Study quality assessment using Newcastle-Ottawa Quality Assessment Forms for Cohort Studies. **Note:** ✩- Star for good quality. ✕ - Not stated.

Ten thousand, three hundred and forty-one unique references were identified, and after screening, 17 studies were yielded for inclusion in the final analysis and quality appraisal (Fig. **1**). All 17 studies were retrospective cohort studies, with 2 using propensity-score matching. Data were from Australia (n=9), the US (n =4), Germany (n =2), Canada (n =1) and Korea (n =1). There was a total of 20,419,197 patients with CVD across the 17 studies. Studies investigated heart failure (HF), acute myocardial infarction (AMI), stroke, atrial fibrillation (AF), cardiac arrest, and general CVD, which included primary hypertension, HF, AF, hypotension, and chronic ischemic heart disease (Table **1**). All studies were rated ‘good’ quality according to the Newcastle-Ottawa Quality Assessment for Cohort Study tools (Fig. **2** and Appendix **2**).

Each HFRS category was reported inconsistently between studies. All 17 studies described the prevalence of each HFRS category, 10 described the age distribution within each HFRS risk group, 10 described the baseline clinical characteristics for each HFRS risk group, and 13 studies calculated adjusted odds ratio (aOR) for mortality. Six studies classified an HFRS ≥ 5 as frail, combining the intermediate and high frailty groups, and 1 study had a no-risk (HFRS=0) category (Table **1**).

**Fig. (3):**
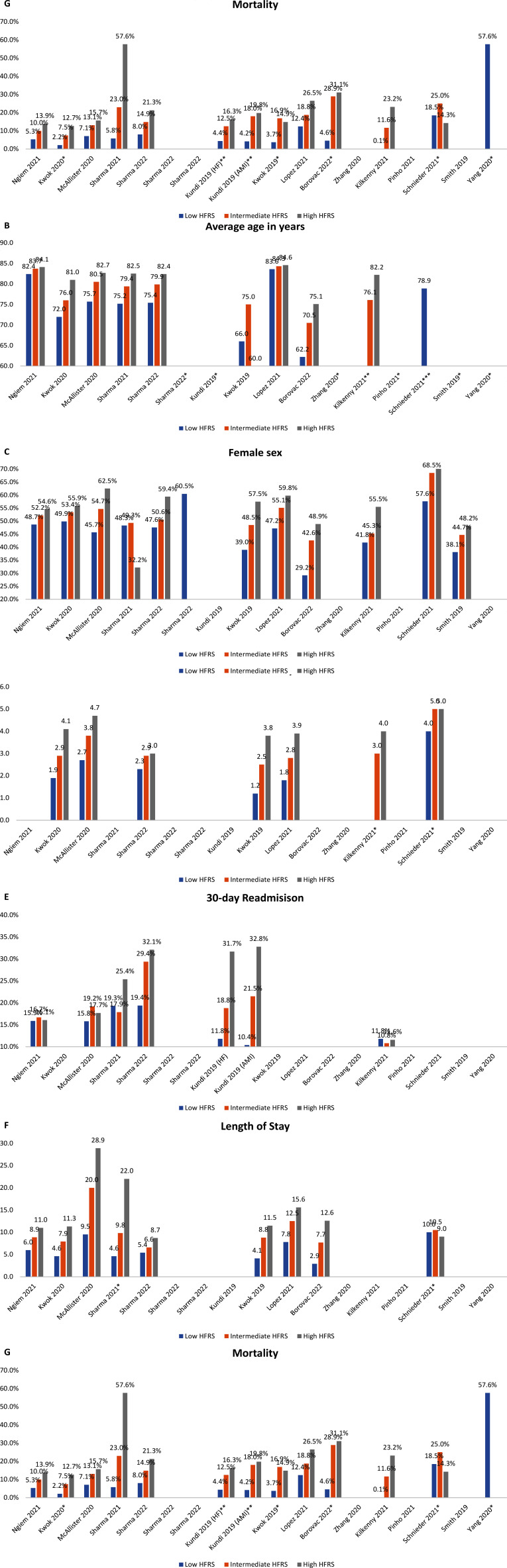
Selected study results: **A.** Prevalence, **B.** Average Age, **C.** Female sex, **D.** Charlson Comorbidity Score, **E.** Readmission, **F.** Length of stay, **G.** Mortality. **Notes:** *Study reported as median and interquartile range.

The prevalence of low, intermediate, and high HFRS ranged from 2.0 to 86.1%, 12.4 to 68.3%, and 0.1 to 40.9%, respectively (Fig. **3A**). The average age of the low, intermediate, and high HFRS ranged from 62.2 to 83.6 years, 70.5 to 84.3, and 75.1 to 84.6, respectively (Fig. **3B**). Increasing frailty score was associated with female sex. The low HFRS group’s prevalence ranged from 29.2 to 57.6% compared to 42.6 to 68.5% for the intermediate HFRS group and 48.2 to 71.4% for the high HFRS group (Fig. **3C**). As measured by the Charlson Comorbidity Index (CCI), the mean comorbidity burden increased with increasing HFRS (Fig. **3D**). Readmission rates at 30 days increased with increasing HFRS category. For the low HFRS group, readmission rates ranged from 2.9 to 6.0%, which differed from 7.7 to 20.0% for the intermediate HRS group and 8.7 to 28.9% for the high HFRS group (Fig. **3E**). This was similar for LOS, which ranged from 2.9 to 6.0 for the low HFRS group, 6.6 to 12.5 for the intermediate HFRS group, and 8.7 to 28.9 for the high HFRS group (Fig. **3F**).

### 3.2 The HFRS and General CVD

There was 1 study that investigated the association of HFRS with a general cohort of patients with CVD aged over 75 years [20]. This study reported specifically on the most prevalent CVD diagnoses, including AF, HF, primary hypertension, hypotension, and chronic ischemic heart disease. Each specific CVD cohort’s age, prevalence, and clinical characteristics were not described. The study reported 30-day mortality for a combined intermediate/high frailty risk group using a bivariate analysis as the attempted regression results were determined to be biased, and no confidence intervals were reported in this study (Fig. **3G**). For the general cohort, the prevalence of low HFRS was 24.6%, intermediate HFRS was 34.5%, and high HFRS was 40.9%. The average age of the low HFRS group was 82.4 years, compared to 83.7 years for the intermediate HFRS group and 84.1 years for the high HFRS risk group. The high frailty group was more likely to be female (54.6%), have a CCI over 2 (86.8%), and increased LOS beyond 10 days (23.9%) compared to their lower frailty risk counterparts. For the combined intermediate/high HFRS group, the aOR of mortality was 1.73 for patients with CVD compared to the low HFRS group. When stratified by diagnosis, aOR of 30-day all-cause mortality using bivariate analysis was 2.15 for primary hypertension, 2.31 for HF, 2.52 for AF, 2.65 for hypotension, and 2.56 for chronic ischemic heart disease compared to the low HFRS group (Table **2**) [20].

### 3.3 The HFRS and HF

Eight studies used the HFRS in patients with HF [20-27]. The prevalence of low HFRS ranged from 24.6 to 86.1%, compared to 12.4 to 47.4% for the intermediate HFRS group and 0.1 to 13.9% for the high HFRS group. The average age of patients with HF and low HFRS ranged from 72.0 to 82.4 years, compared to 76.0 to 80.5 years for intermediate risk and 81.0 to 82.7 years for high HFRS. Increasing female sex was associated with increasing HFRS category. However, 1 study reported decreasing likelihood. Furthermore, increasing HFRS was associated with a higher prevalence of cardiovascular and non-cardiovascular co-morbidities, CCI, and LOS. The aOR of 30-day all-cause mortality ranged from 1.52 (95% confidence interval (CI) 1.50-1.54) to 2.80 (95% CI 2.70-2.90) in patients at intermediate risk and 1.60 (95% CI 1.35-1.90) to 3.50 (95% CI 3.40-3.68) for patients at high risk, compared to their low-risk counterparts (Table **2**).

### 3.4 The HFRS and AMI

Four studies used the HFRS in patients with AMI [21, 28-30]. The prevalence of low HFRS ranged from 49.3 to 86.5%, compared to 13.4 to 36.8% for intermediate HFRS and 0.1 to 13.9% for high HFRS. The age of patients with AMI with a low HFRS ranged from 62.2 to 83.6 years, compared to 70.5 to 84.3 years for patients with an intermediate HFRS and 80.0 to 84.6 years for patients with a high HFRS. Increasing HFRS category was associated with an increased likelihood of female sex, a lower likelihood of white race, and a higher prevalence of cardiovascular and non-cardiovascular co-morbidities. The aOR of mortality ranged from 1.40 (95% CI 1.10-1.79) to 4.02 (95% CI 3.91-4.13) in patients at intermediate risk and 1.58 (95% CI 1.12-2.24) to 4.63 (95% CI 4.36-4.92) for patients at high risk, compared to their low-risk counterparts (Table **2**).

### 3.5 The HFRS and Stroke

Four studies used the HFRS in a cohort with stroke (including ischemic stroke, hemorrhagic stroke, and transient ischemic attack (TIA)) [31-34]. Not all studies reported every outcome. The prevalence of stroke in patients with a low HFRS ranged from 2.0 to 75.1%, compared to 45.0 to 48.0% for patients with an intermediate HFRS and 22.0 to 27.0% for patients with a high HFRS. The ages of patients with stroke with low HFRS were reported in 1 study (78.9 years). The age of patients with stroke with intermediate HFRS ranged from 76.0 to 83.8 years, and high HFRS ranged from 81.0 to 84.2 years. Increasing HFRS category was associated with an increased likelihood of female sex, comorbidities, and LOS. Of the 2 studies that reported aOR of 30-day mortality, this ranged from 1.78 (95% CI 1.33-2.39) to 2.08 (95% CI 1.62-2.67) in patients at intermediate risk and 1.34 (95% CI 1.03-1.75) to 3.55 (95% CI 2.80-4.52) for patients at high risk, in comparison to their low-risk counterparts (Table **2**).

### 3.6 The HFRS and AF

Two studies used the HFRS in patients with AF [20, 35]. Neither study described the age distribution or clinical characteristics in each HFRS category. One study reported non-valvular AF patients exclusively [36]. The prevalence of intermediate HFRS was 14.2%, and high HFRS was 1.6% [36]. This study did not report outcomes stratified by frailty risk category. The study of general CVD reported an aOR of 30-day mortality of 2.52 for combined intermediate/high frailty risk patients compared to their low frailty risk counterparts, with no CI (Table **2**) [20].

### 3.7 The HFRS and Cardiac Arrest

There was a study that used the HFRS in a cohort of patients with cardiac arrest [37]. This study described frailty as HFRS ≥ 5, combining intermediate and high HFRS under this category. The prevalence of low HFRS was 81.4%, and intermediate/high HFRS was 18.6%. This study did not describe age distribution, prevalence, and clinical characteristics for each HFRS category. The aOR of 30-day in-hospital mortality was 2.80 (95% CI 1.52-5.15) for the intermediate/high frailty group, compared to the low-risk group (Table **2**).

## 4 DISCUSSION

Frailty is a significant risk factor for increased hospital LOS, total costs, and adverse health outcomes [4]. The relationship between CVD and frailty is well-established in the literature. However, there is a paucity of data about the use of HFRS in patients with acute and chronic CVD. This is the first systematic review and narrative synthesis about using the HFRS to investigate the prevalence, clinical characteristics, and adverse health outcomes of frailty in patients with CVD. This study has several important findings. Firstly, as defined by the HFRS, intermediate and high frailty risk is present in a significant proportion of patients and across a range of CVD. Patients with an increased risk of frailty are more likely to be older, female sex, non-white race, and have a higher prevalence of co-morbidities. Secondly, increasing frailty is associated with an increased risk of mortality across most CVDs. Finally, the current review indicates that frailty is an essential consideration in patients with CVD. Frail patients with CVD have longer LOS and higher total charges, incurring a great burden on individual patients and health services.

There is no gold standard instrument to assess frailty, despite the abundance of validated tools such as the Frailty Index (FI), Frailty Phenotype (FP), and Clinical Frailty Scale (CFS) [38]. Given technological advancements, electronic healthcare records are more widely implemented, with the advantage of automation intending to streamline and optimize patient care [39]. The HFRS comprises 109 individually weighted ICD-10 codes, sourced entirely from routinely collected health record data from and validated in a UK cohort [14]. The HFRS has been validated in cohorts from Australia, the US, and Germany [25, 28, 33]. The performance of the HFRS is comparable to other digital health record frailty measures and other well-known measures, such as the FI and CFS, in undifferentiated cohorts [14, 40].

Given the heterogeneity in frailty assessment tools deployed in the literature, the reported prevalence of frailty varies [41]. The reported prevalence of frailty in the community is estimated at around 17.9% [41]. However, none of the included studies used the HFRS [41]. This present systematic review estimates the prevalence of frailty as defined by the HFRS in CVD to range between 12.4-48.0% for intermediate risk and 0.1-40.9% for high risk. The wide range in prevalence could be due to a variety of reasons. Firstly, it could be due to the specific limitations of individual studies, such as cohort selection. Most frailty studies have a minimum age cut-off of 65 or 75 years, yet this review found that some studies included all adult hospitalization with no age restrictions [20]. As frailty is related to aging, studies adopting age restrictions would have a more significant proportion of patients at risk of frailty, rendering them less generalizable to the entire population [4, 41, 42]. Secondly, it could be due to the specific CVD being investigated. This is demonstrated by the reported prevalence values being consistent across studies investigating the same CVD. However, studies vary in participant inclusion criteria.

The impact of frailty on outcomes varies with frailty measure, study setting, and length of follow-up [22]. Frailty and CVD share a bidirectional relationship [43]. Frailty increases the odds of CVD by 35% [43], and CVD increases the odds of frailty [43]. The positive correlation is observed using most frailty measures, including the HFRS, which performs as well as most frailty and comorbidity tools as determined by the area under receiver operating curves [14, 40, 44]. There are multiple underlying mechanisms linking CVD and frailty. Briefly, frailty and CVD are age-related conditions that cause a pro-inflammatory state, demonstrated by increased levels of pro-inflammatory cytokines and immune markers, such as interleukin 6 and C-reactive protein [9]. Increased inflammation perpetuates a ‘cycle of frailty’ coined by *Xue et al.*, driving sarcopenia and leading to adverse health outcomes [45]. In the context of CVD, inflammation drives atherosclerosis and plaque instability, increasing the likelihood of plaque rupture and associated complications [46]. The increasing prevalence of CVD and frailty have led to them becoming major public health priorities [47].

The present study found that increasing HFRS was generally associated with female sex, non-white race, and increased prevalence of comorbidities, which is consistent with the wider literature [48-50]. There are a variety of complex factors underpinning these relationships. The literature demonstrates that the female sex is associated with a higher percentage of body fat, predisposing it to the socio-economic and biological factors of frailty [51, 52]. Non-white race is also associated with the development of frailty [53, 54]. The association remains despite adjustment for age, sex, and socio-economic factors. However, the mechanisms behind this are poorly understood, with conflicting results between studies [53, 54]. One of the strongest associations with frailty is comorbidity burden, given their correlation with age [55]. However, both are distinct entities, despite both terms commonly used interchangeably. The normal physiological process of aging and its interaction with diseases leads to the development of frailty, whereas comorbidities play a distinct role in introducing the aging patient to acute stressors, leading to a rapid decline in functional abilities in patients with frailty, which renders them increasingly dependent [55]. It is important to note that patients with increased HFRS are inherently more likely to be more comorbid, as the HFRS was formed as a cumulative deficit model of 109 comorbidities [14].

The present study found that increasing HFRS category was associated with increased odds of mortality for patients with CVD, supporting the previous literature [9, 41, 43, 47]. Frailty and CVD are linked mechanistically through individual cardiovascular conditions. For instance, an aging myocardium is more susceptible to alterations of the electrophysiology of the heart, leading to arrhythmia such as AF, which in turn is an independent predictor of mortality in various conditions [56-58]. AF also predisposes patients to a higher risk of other CVDs, such as stroke, HF, and AMI [59]. Therefore, comorbidities that can contribute to frailty can accumulate and lead to devastating outcomes. The HFRS was derived from patients from an undifferentiated cohort [14]. The receiver operating curves for the HFRS were 0.60 for predicting 30-day mortality, 0.68 for predicting long hospital stays, and 0.56 for predicting 30-day readmission [14]. The performance of the categorized HFRS overlapped with the FI and FP, and the continuous HFRS showed moderate agreement with Rockwood’s FI [14]. Studies using the HFRS in non-CVD-specific cohorts also demonstrate increased odds of mortality with increasing HFRS, with some studies comparing the HFRS to other frailty measures demonstrating comparable performance [60, 61].

Given the increasing age of the population, there is a rising prevalence of CVD and frailty. Hence, this study has several clinical implications. There is a drive toward the use of digital systems in certain areas of the world that facilitate electronic health record data [42, 62, 63]. This drive increases the demand for the automation of processes to improve the efficiency of healthcare services, the accuracy of patient records, and the identification of at-risk individuals based on their past medical history and risk factors [19]. Frailty is a reversible state [13, 38, 64]. The progression of frailty can be affected by early identification, assessment, and intervention [13, 38, 64]. In the context of CVD, cardiologists could do this to improve the prognosis of cardiovascular risk factors and optimize CVD management, though the possible intervention outcomes have not been explored. However, there is a clinical dilemma that is present in older patients diagnosed with CVD. Medications used to treat cardiovascular conditions may be contra-indicated in the frail elderly on the basis of risk-benefit assessment and risks of polypharmacy [65]. Furthermore, the implementation of technology in healthcare infrastructure could benefit frail patients beyond the identification of frailty. For instance, implementing telemedicine services could improve access to care for frail patients once frailty has been identified, allowing for more regular follow-up and personalized care [66, 67].

Future research could address several areas. Firstly, research utilizing the HFRS in patients with CVD should report the baseline clinical characteristics of patients belonging to each HFRS risk group. These groups should be the same as outlined in the development study, being low (<5), intermediate (5-15), and high (>15) HFRS. This would allow comparison between studies and future pooled analysis to determine the overall effect of the HFRS on adverse outcomes of patients with CVD. Secondly, research should address the underlying reasons and potential solutions behind poor outcomes of patients with frailty exhibiting particular demographic and socio-economic factors such as female sex, ethnicity, and household income. Finally, further research could investigate the utility of the HFRS in a cohort with different CVDs to investigate the impact of frailty on the type of CVD a patient is likely to present with and their associated outcomes. The authors have already completed some work to investigate the causes, characteristics, and outcomes of patients presenting secondary with CVD, stratified by their frailty status.

This systematic review has multiple strengths. Firstly, this review followed PRISMA guidelines, ensuring the review was conducted robustly. Secondly, abstracts, full-text screening, and data extraction were conducted independently by two reviewers to minimize bias. Thirdly, the search strategy was broad, as all synonyms for each search term were used. This allowed for the inclusion of most papers on the topic of CVD and frailty, allowing authors to screen for the studies that included the HFRS specifically. Experts in cardiology, epidemiology, and systematic reviews verified the strategy. Finally, all major databases, such as MEDLINE, EMBASE, and WOS, were searched, ensuring the review was comprehensive.

There are several limitations to this systematic review. There was heterogeneity between the focus, design, and reporting of studies. Not all studies described the prevalence or clinical characteristics of each HFRS cohort. Some studies combined intermediate and high frailty groups or created an HFRS 0 group. These factors had direct implications, making comparisons between studies and adjusted analysis difficult due to the different reference groups. Furthermore, studies used different covariates for the multivariable analyses, making comparisons less valid. Again, there was a selection bias inherent to the use of the HFRS. Studies in this review were from countries that have transitioned to electronic health record-keeping and hold registries. Therefore, this review cannot be generalized to healthcare systems that cannot utilize the ICD coding or digital systems.

## CONCLUSION

In conclusion, this systematic review suggests that the HFRS formed entirely from ICD-10 codes, identifies a high-risk phenotype that is associated with mortality in patients with CVD. Increased HFRS is associated with increased age, female sex, non-white racial groups, and increased odds of 30-day all-cause mortality amongst most admission CVD diagnoses. As the burden of frailty and CVD increases and healthcare records transition electronically, the HFRS proves to be a useful tool to stratify frailty risk. Early identification of frailty risk using the HFRS may allow healthcare professionals to optimize cardiovascular risk factors and prevent the downstream adverse outcomes of frailty. Some research suggests that the HFRS performs comparably to other frailty measures in predicting adverse outcomes. Future work should assess the performance of HFRS in identifying high-risk patients in comparison to other contemporary measures of frailty in CVD cohorts.

## Figures and Tables

**Appendix 1:** Example search strategy.

**Search**	**Query**	**Results**
S1	MH Cardiovascular diseases+	2,654,667
S2	MH Cerebrovascular disorders+	415,585
S3	MH Peripheral vascular diseases+	57,271
S4	MH Heart disease risk factors+	4,949
S5	MH Pulmonary embolism+	42,580
S6	TI CVD OR AB CVD	47,631
S7	TI (((Coronary OR Cardiovascular OR Ischaem* OR Ischem* OR Heart OR Myocard* OR Cerebrovascular OR peripheral) N2 (Disease* OR condition* OR disorder*))) OR AB (((Coronary OR Cardiovascular OR Ischaem* OR Ischem* OR Heart OR Myocard* OR Cerebrovascular OR peripheral) N2 (Disease*)))	571,081
S8	TI Atherosclero* OR AB Atherosclero*	166,821
S9	TI Arrythmia* OR AB Arrythmia*	924
S10	TI (((Atrial OR Venric*) N2 (Flutter* or Fib*))) OR AB (((Atrial OR Venric*) N2 (Flutter* or Fib*)))	88,985
S11	TI Arterio* OR AB Arterio*	135,877
S12	TI (((Heart* OR cardiac* OR myocard*) N2 (failure OR insufficien*))) OR AB (((Heart* OR cardiac* OR myocard*) N2 (failure OR insufficien*)))	218,221
S13	TI CCF OR AB CCF	2,046
S14	TI CHF OR AB CHF	15,455
S15	TI (((myocard* OR heart* OR cardiac*) N2 (infarct* OR attack* OR arrest* OR sudden))) OR AB (((myocard* OR heart* OR cardiac*) N2 (infarct* OR attack* OR arrest* OR sudden)))	281,950
S16	TI IHD OR AB IHD	6,532
S17	TI (Cerebrovascular N2 (incident* OR accident*)) OR AB (Cerebrovascular N2 (incident* OR accident*))	7,898
S18	TI CVA OR AB CVA	3,301
S19	TI CVI OR AB CVI	3,386
S20	TI (((Pulm* OR Venous OR Vein OR Art*) N2 (Embol* OR Thromb*))) OR AB (((Pulm* OR Venous OR Vein OR Art*) N2 (Embol* OR Thromb*)))	163,207
S21	TI Claudica* OR AB Claudica*	11,703
S22	TI (((Peripheral OR Limb OR Vascular) N2 (Ischaem* OR Ischem*))) OR AB (((Peripheral OR Limb OR Vascular) N2 (Ischaem* OR Ischem*)))	19,914
S23	TI Stroke* OR AB Stroke*	295,706
S24	S1 OR S2 OR S3 OR S4 OR S5 OR S6 OR S7 OR S8 OR S9 OR S10 OR S11 OR S12 OR S13 OR S14 OR S15 OR S16 OR S17 OR S18 OR S19 OR S20 OR S21 OR S22 OR S23	3,149,197
S25	MM Frailty	6,817
S26	AB Frail* OR TI Frail*	30,965
S27	AB “HFRS” OR TI “HFRS”	1,558
S28	AB HFRS OR TI HFRS	2,594
S29	AB Gilbert* N2 Score OR TI Gilbert* N2 Score	28
S30	S25 OR S26 OR S27 OR S28 OR S29	33,796
S31	MH Animals+	25,862,539
S32	MH Humans	20,806,911
S33	S31 NOT S32	5,055,637
S34	S24 AND S30	5,281
S35	S34 NOT S33	5,241

**Note:** *An asterisk denotes a wildcard symbol, broadening the search term.

+A plus denotes Major Exact Subject Heading search, broadening the search by including all citations with a specific keyword.

**Appendix 2:** Study quality assessment using newcastle-ottawa quality assessment forms for cohort studies.

**Study**	**Representativeness of the Exposed Cohort**	**Selection of the Non-Exposed Cohort**	**Ascertainment of Exposure**	**Demonstration that Outcome of Interest was Not Present at Start of Study**	**Comparability of Cohorts on the Basis of the Design or Analysis Controlled for Confounders**	**Assessment of Outcome**	**Was Follow-up long Enough for Outcomes to Occur**	**Adequacy of Follow-up of Cohorts**	**Quality Score**
Kundi 2019	Truly representative✩	Drawn from the same community as the exposed cohort✩	Secure record✩	No	Study adjusted for: age, sex, race, and comorbidities ✩	Record linkage✩	Yes: 30 day all-cause mortality to assess outcomes after acute presentation✩	Complete follow-up✩	Good
Kwok 2019	Truly representative✩	Drawn from the same community as the exposed cohort✩	Secure record✩	No	Study adjusted for: adjusted for patient demographics, patient co-morbidities, and hospital characteristics ✩	Record linkage✩	Yes: 30 day in-hospital all-cause mortality in same admission episode✩	Complete follow-up✩	Good
Smith 2019	Truly representative✩	Drawn from the same community as the exposed cohort✩	Secure record✩	No	Regression model outcome was developed by including all variables with a *P*-value <0.20 on univariate analysis and then omitting non-significant variables. However exact variables not described.	Record linkage✩	Yes: In-hospital mortality in same episode✩	Complete follow-up✩	Good
Kwok 2020	Truly representative✩	Drawn from the same community as the exposed cohort✩	Secure record✩	No	Study adjusted for: adjusted for patient demographics, patient co-morbidities, and hospital characteristics ✩	Record linkage✩	Yes: 30 day in-hospital all-cause mortality in same admission episode✩	Complete follow-up✩	Good
McAllister 2020	Truly representative✩	Drawn from the same community as the exposed cohort✩	Secure record✩	No	Study adjusted for: age, sex, Charlson Score and LACE score ✩	Record linkage✩	Yes: 30-day and 90-day mortality✩	Complete follow-up✩	Good
Yang 2020	Truly representative✩	Drawn from the same community as the exposed cohort✩	Secure record✩	No	Study adjusted for: Age, gender, heart failure, hypertension, diabetes mellitus, previous, myocardial infarction, peripheral artery disease, economic status, CHA2DS2‐VASc, and HAS‐BLED score.	Record linkage✩	Yes: all-cause mortality until the study endpoint.	Complete follow-up✩	Good
Zhang 2020	Truly representative✩	Drawn from the same community as the exposed cohort✩	Secure record✩	No	Study adjusted for: Prior hospital admission, age, sex, place of birth, background, hospital transfer, in-hospital stroke, ability to walk on admission, history of stroke, socioeconomic status, and comorbidities that were present in more than 10% of the cohort.	Record linkage✩	Yes: all-cause mortality up to 180 days after admission✩	Complete follow-up✩	Good
Kilkenny 2021	Truly representative✩	Drawn from the same community as the exposed cohort✩	Secure record✩	No	Study adjusted for: Age, sex, socioeconomic position, stroke type, and Australian born for mortality outcomes. ✩	Record linkage✩	Yes: 30-day and 90-day all-cause mortality✩	Complete follow-up✩	Good
Lopez 2021	Truly representative✩	Drawn from the same community as the exposed cohort✩	Secure record✩	No	Study adjusted for: Year, age, sex and, number of medicines, Charlson comorbidity index and congestive heart failure, peripheral vascular disease, cerebrovascular disease, dementia, chronic pulmonary disease, rheumatologic disease, peptic ulcer disease, mild liver disease, diabetes hemiplegia/paraplegia, renal disease and cancer.	Record linkage✩	Yes: 30-day mortality✩	Complete follow-up✩	Good
Nghiem 2021	Truly representative✩	Drawn from the same community as the exposed cohort✩	Secure record✩	No	Study adjusted for: Age, sex, ethnicity, marital status and socio-economic status, private hospital insurance, intensive care unit admission, Charlson score and length of stay. ✩	Record linkage✩	Yes: 30-day mortality ✩	Complete follow-up✩	Good
Pinho 2021	Truly representative✩	Drawn from the same community as the exposed cohort✩	Secure record✩	No	All variables significantly associated with favourable outcome in the univariate analysis were included in a multi- variable binary logistic regression analysis and the aOR and 95% CI. However exact variables not described. ✩	Record linkage✩	Yes: 3-month outcomes following acute admission✩	Complete follow-up✩	Good
Schnieder 2021	Truly representative✩	Drawn from the same community as the exposed cohort✩	Secure record✩	No	Univariable logistic regression models used to identify factors associated with a statistical probability (*p* < 0.001) on mortality and then included into the multivariable logistic regression model. However exact variables not described. ✩	Record linkage✩	Yes: 90-day mortality following admission ✩	Complete follow-up✩	Good
Sharma 2021	Truly representative✩	Drawn from the same community as the exposed cohort✩	Secure record✩	No	Study adjusted for: Age, sex, CCI, creatinine, troponin, brain natriuretic peptide (BNP), albumin and c-reactive protein (CRP) levels. ✩	Record linkage✩	Yes: 30-day and 180-day all-cause mortality.	Complete follow-up✩	Good
Borovac 2022	Truly representative✩	Drawn from the same community as the exposed cohort✩	Secure record✩	No	Study adjusted for: Age, sex, race, weekend or elective admission, stay, admission year, insurer, income, hypertension, dyslipidemia, diabetes, peripheral artery disease, CAD, previous MI, HF, previous PCI, previous CABG, previous stroke, AF, valvulopathy, lung disease, renal failure, liver disease, anemia, metastatic cancer, coagulopathy, thrombocytopenia, hospital bed size/ teaching status/ geographic region, cardiogenic shock, in-hospital treatment type and multivessel disease. ✩	Record linkage✩	Yes: 30-day in-hospital all-cause mortality in same admission episode✩	Complete follow-up✩	Good
Sharma 2022	Truly representative✩	Drawn from the same community as the exposed cohort✩	Secure record✩	No	Study adjusted for: Adjusted for age, sex, Charlson score and socioeconomic status ✩	Record linkage✩	Yes: 30-day mortality✩	Complete follow-up✩	Good
Sharma 2022	Truly representative✩	Drawn from the same community as the exposed cohort✩	Secure record✩	No	Study adjusted for: Age, sex, socio-economic status as determined by the IRSD, Charlson index, HFRS, Haemoglobin, Albumin, Creatinine, C-RP, BNP and troponin levels. ✩	Record linkage✩	Yes: 30-day mortality✩	Complete follow-up✩	Good
Sharma 2022	Truly representative✩	Drawn from the same community as the exposed cohort✩	Secure record✩	No	Study did not adjust for covariates	Record linkage✩	Yes: 30-day mortality✩	Complete follow-up✩	Good

**Abbreviations:** LACE - length of stay (L), acuity of admission (A), Charlson comorbidity score (C), and emergency room utilization in the prior 6 months (E). ✩ - Good quality.

**Table 1:** Selected study characteristics and results.

**Study**	**Study design**	**Country**	**Study Period**	**Sample Size**	**Participant Inclusion Criteria**
***General CVD Cohort***					
Nghiem 2021^a^	Retrospective cohort	Australia	2012-2015	115,946	Over 75 with CVD (primary hypertension, HF, AF, hypotension, chronic ischaemic heart disease)
***HF Cohort***					
Kwok 2020	Retrospective cohort	USA	2004-2014	11,626,400	Adult (>18) hospitalisations for HF
McAllister 2020	Retrospective cohort	Canada	2004-2016	26,626	First time adult (>18) hospitalisation for HF
Sharma 2021^a^	Retrospective cohort;	Australia	2015-2019	3,706	First time adult (>18) hospitalisation for HF
Sharma 2022	Retrospective cohort	Australia	2013-2020	5,735	Older than 75 with HF
Sharma 2022^a,b^	Retrospective propensity-matched cohort	Australia	2013-2020	5,734	Adults (>18) hospitalised with HF
Sharma 2022^a,c^	Retrospective propensity-matched cohort	Australia	2013-2019	4,913	Adults (>18) hospitalised with HF
***AMI and HF Cohorts***					
Kundi 2019	Retrospective cohort	USA	2016	785,127	Medicare fee-for-service beneficiaries 65 years and older admitted with AMI, HF or pneumonia
***AMI Cohorts***					
Kwok 2019	Retrospective cohort	USA	2004-2014	7,393,268	Adult (>18) hospitalisations for ACS
Lopez 2021	Retrospective cohort	USA	2003-2008	2,761	Over 80 hospitalised with AMI
Borovac 2022	Retrospective cohort	USA	2015-2017	429,070	Adults (>18) hospitalised with STEMI
***Stroke Cohorts***					
Zhang 2020	Retrospective cohort	Australia	2009-2013	15,482	Adults (>18) with intracerebral haemorrhage
Kilkenny 2021^d^	Retrospective cohort	Australia	2009-2013	15,468	Adults (>18) with stroke and TIA
Pinho 2021^e^	Retrospective cohort	Germany	2012-2017	489	Hospitalisations for acute ischemic stroke receiving endovascular treatment
Schnieder 2021^a^	Retrospective cohort	Germany	2015-2019	318	Hospitalisations for acute ischemic stroke receiving endovascular treatment for large vessel occlusion stroke
***Cardiac Arrest Cohort***					
Smith 2019^a^	Retrospective cohort	Australia	2008-2017	388	All in-hospital cardiac arrest involving rapid response team attendance
***AF Cohort***					
Yang 2020^f^	Retrospective cohort	Korea	2005-2015	262,987	Adults (>18) with non-valvular AF

**Note :**^a^ Study defined frailty as a HFRS ≥ 5.

^b^ Cohorts separated into 4 groups: frail and non-frail with and without specific pharmacotherapy.

^c^ Study investigated whether admission under general medicine or cardiology specifically impacted outcomes.

^d^ Study included a no risk frailty group (HFRS=0).

^e^ Study divided outcomes into favourable and poor at 3-months.

^f^ Study investigated different clinical management pathways (ABC and no ABC) and effect of HFRS specifically.

**Abbreviations:** aOR – Adjusted odds ratio; AF – Atrial Fibrillation; AMI – Acute Myocardial Infarction; CVD – Cardiovascular Disease; CI – Confidence Interval; HF – Heart Failure; HFRS – Hospital Frailty Risk Score; NSTEMI – Non ST-Elevation Myocardial Infarction; STEMI – ST-Elevation Myocardial Infarction; TIA – Transient Ischaemic Attack.

**Table 2:** Selected study results.

**Study**	**HFRS Category**	**Prevalence by HFRS Category, %**	**Age in Years**	**Clinical Characteristics of High HFRS Category, %**	**Odds of Mortality with Low HFRS Reference Group, aOR (CI)**
***General CVD Cohort***					
Nghiem 2021^a^	Low	24.6	82.4 ± 5.2	High HFRS group were more likely to be female (54.6), have a Charlson score above 2 (86.8), have a length of stay beyond 10 days (32.9).	Bivariate analysis of 30-day mortality with high relation coefficient (0.92, *p<*0.01) Intermediate/High: 1.73 for all CVD, 2.15 for primary hypertension, 2.31 for HF, 2.52 for AF, 2.65 for hypotension and 2.56 for chronic ischaemic heart disease.
	Intermediate	34.5	83.7 ± 5.5		
	High	40.9	84.1 ± 5.5		
***HF Cohort***					
Kwok 2020	Low	80.0	72.0 ± 15.0	Greater proportion of female patients in intermediate (53.4) and high (55.9) groups. Greater prevalence of previous stroke (17.2) and peripheral vascular disease (15.4), for high frailty risk group.	Intermediate: 1.52 [1.50-1.54] High: 1.60 [1.35-1.90] All *p*<0.001
	Intermediate	19.9	76.0 ± 13.0		
	High	0.1	81.0 ± 11.0		
McAllister 2020	Low	66.6	75.7 ± 13.0	Higher HFRS group were more likely to have higher Charlson, female sex and decreased likelihood of baseline independence, more prior emergency department visits and more prior hospitalizations.	Results not presented as odds ratios. 30-day mortality presented as crude rates.
	Intermediate	26.4	80.5 ± 11.6		
	High	6.9	82.7 ± 10.3		
Sharma 2021^a^	Low	76.4	75.2 ±1 4.4	High HFRS group were more likely to be female (67.8), have a higher Charlson score (3.9).	30-day all-cause mortality: Intermediate/High: 4.12, [2.71–6.27], *p* < 0.001
	Intermediate	22.0	79.4 ± 12.3		
	High	1.6	82.5 ± 11.1		
Sharma 2022	Low	86.1	75.4 ± 14.3	High HFRS group were less likely to be female (40.6) and more likely to have a higher mean Charlson score (3.0) and increased length of stay (8.7).	30-day all-cause mortality: Intermediate: 1.52 [1.20-1.93] High: 2.09 [1.21-3.60]
	Intermediate	12.4	79.9 ± 11.3		
	High	1.6	82.4 ± 1.6		
Sharma 2022^a,b^	Low	7.5.5	Characteristics for each frailty category not described.	Characteristics for each frailty category not described.	In-hospital all-cause mortality of frail patients receiving pharmacotherapy: Intermediate/High: 0.20 [0.15-0.27]
	Intermediate/High	24.5			
Sharma 2022^a,c^	Low	76.2	Characteristics for each frailty category not described.	Characteristics for each frailty category not described.	Outcomes for each frailty category not compared to lower frailty category.
	Intermediate/High	23.8			
***AMI and HF Cohorts***					
Kundi 2019	Low	49.3 for AMI, 27.5 for HF	Characteristics for each frailty category not described. Mean age of AMI cohort was 77.4±8.7, and HF cohort was 80.1±9.0	Greater proportion of female patients in intermediate (48.5%) and high (47.5%) group. Those with higher HF RS had greater prevalence of HF (4.5%), AF (30.5%), previous stroke (19.3%), peripheral vascular disorders (15.7%), chronic lung disease (20.6%), renal failure (50.9%), liver failure (1.9%), and dementia (60.3%).	30-day in-hospital all-cause mortality: Intermediate: 3.73 [3.66-3.80] High: 2.57 [2.18-3.04] All *p*<0.001.
	Intermediate	36.8 for AMI, 47.4 for HF			
	High	13.9 for AMI, 25.0 for HF			
***AMI cohorts***					
Kwok 2019	Low	86.5	66±14	Greater proportion of female patients in intermediate (48.5%) and high (47.5%) group. Those with higher HF RS had greater prevalence of HF (4.5%), AF (30.5%), previous stroke (19.3%), peripheral vascular disorders (15.7%), chronic lung disease (20.6%), renal failure (50.9%), liver failure (1.9%), and dementia (60.3%).	30-day in-hospital all-cause mortality: Intermediate: 3.73 [3.66-3.80] High: 2.57 [2.18-3.04] All *p*<0.001
	Intermediate	13.4	75±13		
	High	0.1	80±11		
Lopez 2021	Low	59.1	83.6 ± 2.7	High HFRS group were more likely to be female (59.8), and have history of HF (38.7), cerebrovascular disease (40.9), dementia (35.1), chronic pulmonary disease (23.8), renal disease (23.5), cancer (23.2) and higher Charlson score (3.9).	30-day all-cause mortality with HFRS in model: Intermediate: 1.40 [1.10-1.79] High: 1.58 [1.12-2.24]
	Intermediate	29.0	84.3 ± 2.8		
	High	11.9	84.6 ± 2.7		
Borovac 2022	Low	71.6	62.2 ± 12.9	High HFRS group were more likely to be female (48.9), less likely to be white (69.5), smoke (0.8) and receive PCI (22.6). High HFRS group had lower prevalence of arterial hypertension (29.8) and dyslipidaemia (43.3). High HFRS had a higher prevalence of peripheral artery disease (10.6), AF (27.4), anaemia (40.5) and renal failure (44.9).	Intermediate: 4.02 [3.91-4.13] High: 4.63 [4.36-4.92]
	Intermediate	26.3	70.5 ± 13.7		
	High	2.1	75.1 ± 12.7		
***Stroke cohorts***					
Zhang 2020	Low	25.0	Characteristics for each frailty category not described.	Characteristics for each frailty category not described.	Univariate model hazard of death after intracerebral haemorrhage: Intermediate: 1.18 [0.96-1.44] *p*=0.115 High: 1.70 [1.37-2.10] *p*<0.001 Multivariate model: Intermediate: 1.78 [1.33-2.39] *p*<0.001 High: 1.34 [1.03-1.75] *p*=0.032
	Intermediate	48.0			
	High	27.0			
Kilkenny 2021^d^	No risk (HFRS=0)/ Low risk (HFRS=1-4)	9.0 for no risk, 23.0 for low risk	67.0 (57.4-75.6) for no risk (HFRS=0), 71.5 (60.8-80.5)	High HFRS group were more likely to be female (55.5), suffer severe stroke (16.5) and have more comorbidities, history of stroke and length of stay.	30-day all-cause mortality compared to no-risk group Low: 0.95 [0.70-1.28] Intermediate: 2.08 [1.62-2.67] High: 3.55 [2.80-4.52] 90-day all-cause mortality compared to no-risk group Low: 1.06 [0.82-1.36] Intermediate: 2.29 [1.85-2.84] High: 4.33 [3.41-5.49]
	Intermediate	45.0	76.1 (65.4-83.8)		
	High	22.0	82.2 (74.3-87.5)		
Pinho 2021^e^	Low	2.0	Characteristics for each frailty category not described.	Characteristics for each frailty category not described.	‘Favourable’ 3-month outcome defined by modified Rankin scale of 0-2. High: 0.48 [0.26-0.89] (*p*=0.020)
	Intermediate	68.3			
	High	29.7			
Schnieder 2021^a^	Low	75.1	78.9 (9.6)	High HFRS group were more likely to be female (71.4).	90-day all-cause mortality: High: 1.12 [1.02-1.24] *p*=0.020
	Intermediate	22.7	83.8 (9.6)		
	High	2.2	84.2 (7.2)		
***Cardiac arrest cohort***					
Smith 2019^a^	Low	81.4	Characteristics for each frailty category not described.	Characteristics for each frailty category not described.	In-hospital mortality: Intermediate/High: 2.80 [1.52-5.15] *p*<0.001
	Intermediate/High	18.6			
***AF cohort***					
Yang 2020^f^	Low	84.2	Characteristics for each frailty category not described.	Characteristics for each frailty category not described.	Outcomes for each frailty category not described.
	Intermediate	14.2			
	High	1.6			

**Note:** ^a^ Reported as per study as median with IQR, or mean with SD.

^b^ Study defined frailty as a HFRS ≥ 5.

^c^ Cohorts separated into 4 groups: frail and non-frail with and without specific pharmacotherapy.

^d^ Study investigated whether admission under general medicine or cardiology specifically impacted outcomes.

^e^ Study included a no risk frailty group (HFRS=0).

^f^ Study divided outcomes into favourable and poor at 3-months.

^g^ Study investigated different clinical management pathways (ABC and no ABC) and effect of HFRS specifically.

**Abbreviations:** aOR – Adjusted odds ratio; AF – Atrial Fibrillation; AMI – Acute Myocardial Infarction; CVD – Cardiovascular Disease; CI – Confidence Interval; HF – Heart Failure; HFRS – Hospital Frailty Risk Score; NSTEMI – Non ST-Elevation Myocardial Infarction; STEMI – ST-Elevation Myocardial Infarction; TIA – Transient Ischaemic Attack.

## LIST OF ABBREVIATIONS

CVD: Cardiovascular Disease
NOS: Newcastle-Ottawa Scale
HF: Heart Failure
LOS: Length of Stay
ICD: International Classification of Diseases

## STANDARDS OF REPORTING

PRISMA guidelines and methodology were followed.
